# The Draft Assembly of the Radically Organized *Stylonychia lemnae* Macronuclear Genome

**DOI:** 10.1093/gbe/evu139

**Published:** 2014-06-20

**Authors:** Samuel H. Aeschlimann, Franziska Jönsson, Jan Postberg, Nicholas A. Stover, Robert L. Petera, Hans-Joachim Lipps, Mariusz Nowacki, Estienne C. Swart

**Affiliations:** ^1^Institute of Cell Biology, University of Bern, Switzerland; ^2^Centre for Biological Research and Education (ZBAF), Institute of Cell Biology, Witten/Herdecke University, Wuppertal, Germany; ^3^Department of Neonatology, HELIOS Children’s Hospital, Witten/Herdecke University, Wuppertal, Germany; ^4^Biology Department, Bradley University

**Keywords:** macronuclear genome, nanochromosome, genome rearrangement, histone variant, chromosome copy number, alternative fragmentation

## Abstract

*Stylonychia lemnae* is a classical model single-celled eukaryote, and a quintessential ciliate typified by dimorphic nuclei: A small, germline micronucleus and a massive, vegetative macronucleus. The genome within *Stylonychia*’s macronucleus has a very unusual architecture, comprised variably and highly amplified “nanochromosomes,” each usually encoding a single gene with a minimal amount of surrounding noncoding DNA. As only a tiny fraction of the *Stylonychia* genes has been sequenced, and to promote research using this organism, we sequenced its macronuclear genome. We report the analysis of the 50.2-Mb draft *S. lemnae* macronuclear genome assembly, containing in excess of 16,000 complete nanochromosomes, assembled as less than 20,000 contigs. We found considerable conservation of fundamental genomic properties between *S. lemnae* and its close relative, *Oxytricha trifallax*, including nanochromosomal gene synteny, alternative fragmentation, and copy number. Protein domain searches in *Stylonychia* revealed two new telomere-binding protein homologs and the presence of linker histones. Among the diverse histone variants of *S. lemnae* and *O. trifallax*, we found divergent, coexpressed variants corresponding to four of the five core nucleosomal proteins (H1.2, H2A.6, H2B.4, and H3.7) suggesting that these ciliates may possess specialized nucleosomes involved in genome processing during nuclear differentiation. The assembly of the *S. lemnae* macronuclear genome demonstrates that largely complete, well-assembled highly fragmented genomes of similar size and complexity may be produced from one library and lane of Illumina HiSeq 2000 shotgun sequencing. The provision of the *S. lemnae* macronuclear genome sets the stage for future detailed experimental studies of chromatin-mediated, RNA-guided developmental genome rearrangements.

## Introduction

**Fig. 1.—:**
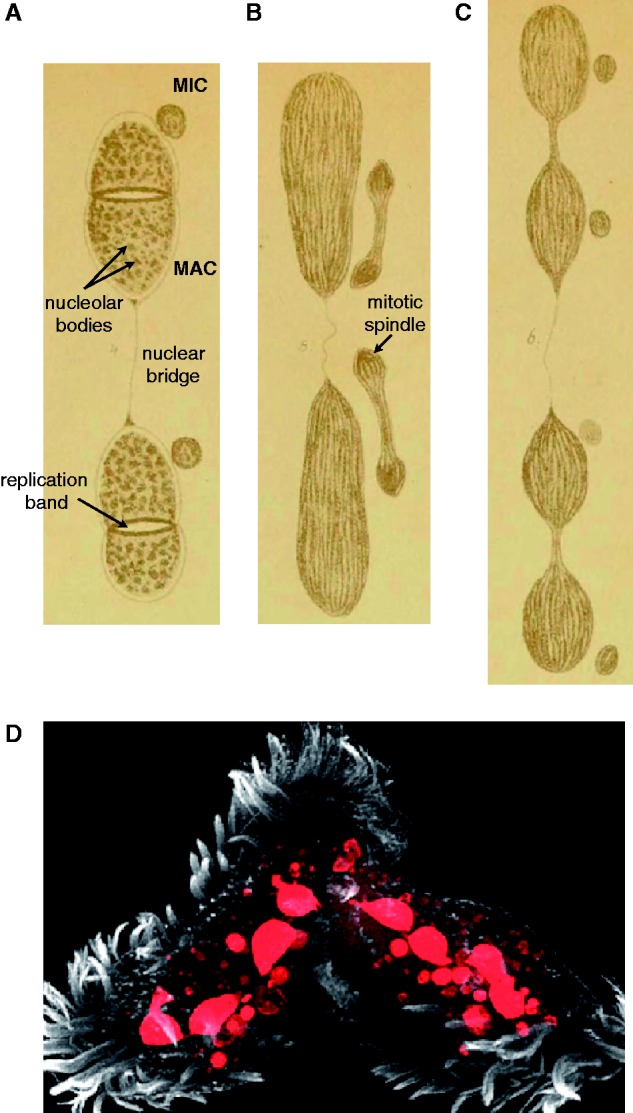
*Stylonychia* macronuclei. (*A–C*) Illustrations of successive stages of *Stylonychia* nuclear division during cellular replication, modified from (Bütschli 1876). Replication bands are responsible for DNA synthesis in spirotrichous ciliates (Gall 1959), including *Styonychia*, and sweep through the MAC during asexually division (Ammermann 1971). The granular structure of macronuclei, due to nucleolar bodies (Postberg et al. 2006) is also shown. There is currently no indication of a classical spindle in macronuclei (Ammermann 1971). (*D*) A pair of conjugating *S*. *lemnae* cells containing a mixture of old fragmenting macronuclei, new macronuclei, and micronuclei (DAPI staining in red; overlaid on a micrograph of the cells) illustrating the complexity of nuclear organization and development.

As is characteristic of ciliates, *Stylonychia lemnae* possesses both a macronucleus (MAC), specialized for gene expression, and a micronucleus (MIC), containing the germline genome that permits recombination and transmission of genetic information across sexual generations (Prescott 1994) (fig. 1). As a genus, *Stylonychia* has long and rich history as a subject for studies of nuclear organization and development, chromosomes and chromatin, and telomere biology and genome rearrangement (reviewed in Prescott 1994, 2000; Fuhrmann et al. 2013). Among the first records of chromosomes and the mitotic spindle were detailed drawings of micronuclei from *Stylonychia* species published by Bütschli (1876) (fig. 1*B*). The discovery of a large DNA loss (over 90%) in *S. lemnae*’s developing MAC following polyploidization (Ammermann 1968) spurred the studies of genome reduction and reorganization in ciliates (Prescott 1994). Subsequently *S. lemnae* (which, for simplicity’s sake, we refer to as *Stylonychia* henceforth) has been extensively used as a model unicellular organism to study the regulation of telomere structure (reviewed in Lipps and Rhodes 2009) and chromatin dynamics (Postberg et al. 2008, 2010; Bulic et al. 2013; Forcob et al. 2014) during genome reorganization and macronuclear differentiation. In spite of the success in studying these processes, their analysis has been hampered by the difficulty of manipulating *Stylonychia* by classical genetic means and by the limited availability of sequence information. The provision of an annotated *Stylonychia* draft MAC genome is a significant contribution to addressing the latter problem.

The genomes contained within the *Stylonychia* micro- and macronuclei both have extraordinary architectures, with the former containing elaborately “scrambled” DNA segments which need to be reorganized and joined to form a highly fragmented genome comprised “nanochromosomes.” During sexual development (triggered by conjugation of compatible pairs of cells), a copy of the MIC genome develops into a fresh MAC genome by sophisticated reorganization processes including: 1) The excision of intervening sequences (“internally eliminated sequences,” or IESs) in MAC-destined sequences, 2) unscrambling of MAC-destined DNA, 3) elimination of bulk DNA containing both repetitive and unique DNA, 4) fragmentation of the genome, 5) de novo addition of telomeres, and 6) and amplification of MAC sequences to specific copy numbers (reviewed in Prescott 1994, 2000). The MAC genomes of stichotrichous ciliates (including *S. lemnae* and *Oxytricha trifallax*) are organized as tiny, mostly gene-sized molecules with a minimal amount of subtelomeric noncoding sequence, allowing them to be exploited as natural gene finders for both protein-coding and noncoding RNA genes (Jung et al. 2011; Swart et al. 2013). Mature nanochromosomes are capped on either end by simple telomeric repeats (Oka et al. 1980; Klobutcher et al. 1981; Lipps and Erhardt 1981; Pluta et al. 1982). In stichotrichs, alternative processing of certain developing macronuclear DNA regions generates nanochromosome isoforms (Herrick et al. 1987). In *O. trifallax* (henceforth, *Oxytricha*), approximately 10% of nanochromosomes have more than one site of telomere addition and give rise to one or a few isoforms, typically with intact genes (Swart et al. 2013).

Macronuclear DNA giving rise to nanochromosomes is variably amplified in two successive rounds (Ammermann 1971; Ammermann et al. 1974) during development, resulting in thousands of copies of each nanochromosome (∼15,000 copies in *S. lemnae* [Steinbrück 1983] and ∼1,900 copies in *O. trifallax* [Prescott 1994] on average). In *Stylonychia* and *Oxytricha*, the most highly amplified nanochromosome encodes the 18S, 5.8S, and 28S rRNA subunits (Lipps and Steinbruck 1978; Spear 1980; Swanton et al. 1980, 1982). In *Oxytricha*, the copy number of this nanochromosome is approximately 56 times the mean nanochromosome copy number (Swart et al. 2013). The copy number of most *Oxytricha* nanochromosomes lies within approximately a single order of magnitude interval (Swart et al. 2013). Macronuclear division during cellular replication occurs by a process known as amitosis, which results in stochastic segregation between the two resulting nuclei without the aid of a mitotic spindle (reviewed in Prescott 1994). In ciliates the amount of DNA amplification in mature macronuclei is proportional to their cell volume (Taylor and Shuter 1981), which has been hypothesized to reflect the need for increased gene expression as cell size increases (Bell 1988) (the largest ciliates may be as long as a few millimeters [Lynn 2008]). Thus, *S. lemnae* cells (largest dimension 140 μm [Prescott 1994]) were estimated to have approximately 6.8× the macronuclear DNA content of cells from *Oxytricha* species (Prescott 1994) (largest dimension ∼95 μm).

Although the *O. trifallax* MAC genome has recently been published (Swart et al. 2013), there is also considerable interest in obtaining a draft *S. lemnae* MAC genome, both for comparative purposes and to facilitate future detailed experimental studies using *Stylonychia* as a model organism. We show that continued improvements in genome assembly and conventional Illumina sequencing now permit the production of well-assembled, and largely complete draft nanochromosomal MAC genomes with just a single Illumina paired-end (PE) DNA-seq library (∼68× coverage with 90-bp reads). We report the first comparative genomic analyses between two highly fragmented macronuclear genomes, focusing on gene synteny and nanochromosome copy number conservation. We conclude by describing the diversity of histone variants in *Stylonychia*, including a number of unique, highly divergent histone H1, H2A, H2B, and H3 variants. Our discoveries suggest that a wealth of treasures is still waiting to be revealed in the macronuclear genomes of both *Oxytricha* and *Stylonychia*.

## Materials and Methods

### DNA Isolation, Illumina Library Creation, and Sequencing

Illumina shotgun libraries were created for *S. lemnae* cells of lab strain 130c. This strain was created by conjugation of cells from two different mating types derived from two inbred lab strains derived from cells originally collected in Southern Germany (*Stylonychia* strains senescence after some time in laboratory culture and so it is necessary to conjugate the cells on a regular basis [Duerr et al. 2004]). *Stylonychia lemnae* cells were grown and lysed as previously described (Ammermann et al. 1974). After lysis of cells, macronuclei were separated from micronuclei by sieving the cell lysates twice through a 15-µm gauze, collecting the macronuclei on the gauze. Macronuclei were collected by centrifugation (4 °C, 2,400 rpm, 6 min, 4 °C) and then lysed in Karvenoff–Zimm buffer (10 mM Tris; 0.5 M EDTA; 1% SDS; pH 9.5) for 30 min at 65 °C. Proteinase K (0.2 mg/ml) was then added (50 °C, overnight) followed by RNase digestion (1 h, 37 °C). After phenol/chloroform extraction, DNA was dialyzed against 0.1× Tris–ethylenediaminetetraacetic acid (24 h, 4 °C) followed by ethanol precipitation.

The Beijing Genome Institute produced standard Illumina genomic libraries from macronuclear DNA fragmented to specific size ranges by a Covaris E220 sonicator, followed by size selection of the library DNA fragments during agarose gel electrophoresis. Sequencing was performed on a HiSeq 2000 sequencer.

### Choice of Illumina Library for Assembly

Four libraries with different PE length distributions were created from *Stylonychia* macronuclear DNA (distributions of PE read lengths are shown in supplementary fig. S3, Supplementary Material online). We define the best assembly as the assembly which maximizes the ratio of total nanochromosomes to total contigs. Irrespective of the assembler used, the best assemblies were typically produced from the shortest PE library (e.g., table 1). Combining this library with other libraries usually produced worse assemblies. We therefore focused our efforts on optimizing the assembly with this library alone.

**Table 1:** SPAdes Assemblies of different *Stylonychia* MAC DNA Libraries

Property	Library 24	Library 25	Library 26	Library 27
Assembly size (Mb)	52.8	47.9	50.1	49.9
Contigs (*n*)	29,175	23,673	32,495	41,263
Telomeres (*n*)	34,815	13,806	27,912	9,878
Mean contig length (bp)	1,811	2,022	1,542	1,210
Max contig length (bp)	65,401	65,232	65,436	65,079
2-Telomere contigs	16,082	3,822	10,912	2,069
1-Telomere contigs	2,550	6,106	6,028	5,731
0-Telomere contigs	10,543	13,745	15,555	33,463
Total PE read coverage (%)	98.1	91.1	93.0	96.3
Telomeric PE read coverage (%)	92.2	59.5	79.7	62.5

### IDBA-UD/Terminator 2.0 Assembly

Our initial best macronuclear genome assembly for *Stylonychia* with IDBA-UD contained approximately 9,400 nanochromosomes, but most of the contigs in the assembly lacked one or both telomeres (∼29,100).

We were able to significantly improve the *O. trifallax* macronuclear genome assembly by a custom meta-assembly pipeline (which we now call “Terminator 1.0”), but this pipeline was complex and used multiple sequence types. We therefore developed a simplified version of this approach for *S. lemnae* (Terminator 2.0) using Illumina sequence data alone (supplementary fig. S1, Supplementary Material online). We used CAP3 (Huang and Madan 1999) to merge assemblies, as was done for the *Oxytricha* MAC genome assembly, but instead of attempting to prevent collapse of all alleles, we chose a less restrictive assembly criterion: ≥40 bp matches that are ≥97% identity. To extend the contigs we used two different read mappers, SHRiMP 2 (Rumble et al. 2009; David et al. 2011) and smalt (version 0.71; http://www.sanger.ac.uk/resources/software/smalt/, last accessed June 30, 2014). An improvement over the *Oxytricha* pipeline is that we used read pairing information to avoid incorrect contig extension. This extension process is relatively strict, as we only permit an extension if one of the reads in the read pair matches with zero or one substitution to a contig end. We did not include the chimera detection and removal step used in the *Oxytricha* macronuclear genome assembly, since, as judged by visual inspection of *Stylonychia* assemblies, this did not appear to be a significant issue.

In the Terminator 2.0 pipeline, we first extended the contigs with SHRiMP until there were no longer any significant improvements after assembly with CAP3 (at ∼16 iterations). After visual inspection of contigs that failed to be extended by SHRiMP, but were extended by the Geneious read mapper (Kearse et al. 2012), we found that many contigs could still be extended. We found that smalt was capable of mapping additional reads to many of the ends, and so we continued to extend the contigs with smalt. The proportion of nanochromosomes increased only slightly with increasing extensions, and so we chose our final CAP3 meta-assembly as the assembly after ten additional extensions using smalt.

The successive rounds of extension of the contigs using SHRiMP mapping results, followed by CAP3 meta-assembly, increased the total nanochromosome tally by approximately 4,800. This tally was further increased by approximately 1,850 nanochromosomes after a further round of extension using smalt, followed by CAP3 meta-assembly, yielding a total of approximately 16,100 nanochromosomes (table 2).

**Table 2:** Best *Stylonychia* MAC Genome Assemblies

Property	SPAdes (SE)	SPAdes (PE)	Terminator 2.0	Final (SPAdes Polished)
Assembly size (Mb)	52.2	52.8	54.7	50.2
Contigs (*n*)	31,850	29,175	22,758	19,851
Telomeres (*n*)	34,422	34,815	35,961	34,327
Mean contig length (bp)	1,640	1,811	2,404	2,531
Max contig length (bp)	65,404	65,401	65,407	65,401
2-Telomere contigs	14,619	16,082	16,082	16,059
1-Telomere contigs	5,132	2,550	3,683	2,104
0-Telomere contigs	12,099	10,543	2,993	1,688
Total PE read coverage (%)	97.9	98.1	98.2	98.0
Telomeric PE read coverage (%)	90.0	92.2	90.4	91.0

### SPAdes Assembly

SPAdes (2.5.0) (Bankevich et al. 2012) was run with the “careful” option and the BayesHammer error correction algorithm (Nikolenko et al. 2013) on Illumina library 24. CAP3 (Huang and Madan 1999) was used to assemble potential overlapping contigs from the SPAdes assembly, using a coverage length cutoff of 40 bp and a overlap percent identity cutoff of 97. To remove redundant “chaff” contigs from the assembly, BLAT (Kent 2002) was used to map contigs shorter than 500 bp to contigs greater than 500 bp. Contigs shorter than 500 bp were discarded (8,920 in total) if they had matches to the greater than 500 bp contigs with greater than 80% coverage and greater than 90% sequence identity (this had a minimal effect on the assembly completeness: See table 2).

### Assembly Cleanup

Prior to submitting the macronuclear genome to the European Nucleotide Archive, we clipped Illumina adapters at the ends of contigs matching adaptor sequences in the UniVec (ftp://ftp.ncbi.nih.gov/pub/UniVec/UniVec, last accessed June 30, 2014) and EMVec (ftp://ftp.ebi.ac.uk/pub/databases/emvec/emvec.dat.gz, last accessed June 30, 2014) databases (using BLASTN with default parameters; BLAST+ 2.2.26 [Camacho et al. 2009]). We also removed four contigs (Contig8462, Contig1165, Contig12163, and Contig7566) with longer matches to these vectors databases.

The macronuclear isolation protocol used kept DNA contamination from *Stylonychia* mitochondria to a minimum: Only two short (<1,080 bp), telomereless contigs (Contig10464 and Contig843) had substantial TBLASTX matches (*e* value < 1e-3) to the *Oxytricha* mitochondrial genome (Swart et al. 2012). These contigs were removed.

### Genome Assemblies from Other Assemblers

The following additional genome assemblers were tested: ABySS (Simpson et al. 2009), IDBA-UD (Peng et al. 2012), Velvet (Zerbino and Birney 2008), MetaVelvet (Namiki et al. 2012), Minia (Chikhi and Rizk 2013), Mira (Chevreux et al. 1999), and SOAPdenovo (Li et al. 2010). See supplementary table S1, Supplementary Material online, for statistics of the best genome assemblies produced with these assemblers, all using library 24. ABySS (version 1.3.4) was run in default mode with a k-mer size of 31. IDBA-UD (version 1.0.9) was run with the switches “–mink 25 –maxk 89 –step 2.” Velvet was run with k-mer size 31, automatic coverage and cutoff of 10. The Velvet assembly was used as input to MetaVelvet (version 1.2.02) run in default mode. Minia was compiled using the make k = 100 option to include higher k-mer sizes and run with a k-mer size of 31 and minimum abundance of 4. Mira (version 3.4.1.1) was run in accurate mode (job=denovo,genome,accurate,solexa SOLEXA SETTINGS—GE:tismin=100:tismax=200) on a subset of 20 million reads randomly chosen from library 24 by a custom Python script. SOAPdenovo was run in default mode with the multi-kmer switch “-m 63.”

### Validation of Final Genome Assembly

As the mean insert size of library 25 is relatively large (463 bp) compared with the average nanochromosome size, mapped reads from this library provide a manner to generally validate our final assembly. Tolerating no substitutions during read mapping, 98.9% of the mapped reads were properly paired, as defined by the read mapper bwa (Li and Durbin 2009) (i.e., correctly oriented and within a reasonable insert size range; see http://bio-bwa.sourceforge.net/bwa.shtml, last accessed June 30, 2014).

### Gene Prediction

*Stylonychia* gene predictions were produced de novo using AUGUSTUS (version 2.5.5) (Stanke et al. 2006) previously trained on *O. trifallax* (Swart et al. 2013) with the following parameters: “−−species=oxytricha −−UTR=on −−extrinsicCfgFile=install/augustus.2.5.5/config/extrinsic/extrinsic.M.RM.E.W.cfg −−alternatives−from−evidence=true −−genemodel=complete −−codingseq=on.” No RNA-seq data were used to provide additional constraints (hints) for the gene prediction.

Overall, the *Stylonychia* gene prediction statistics (supplementary table S2, Supplementary Material online) are similar to those from *Oxytricha* (supplementary table S3, Supplementary Material online), which is presumably both a consequence of the training on *Oxytricha* data, and the similarity between these organisms. In *Stylonychia* and *Oxytricha* (with RNA-seq “hints” for AUGUSTUS), 73% and 76% of nanochromosomes are predicted to contain a single gene, respectively.

### Protein Annotation

HMMER version 3.1b1 (Eddy 2013) was used to annotate protein domains with the Pfam database (Mistry et al. 2013) (version 26.0). Supplementary data file S1 (stylo_asm1.all.domtable.txt.zip), Supplementary Material online, contains these annotations.

Blast2GO (Conesa et al. 2005; Gotz et al. 2008) (version 2.5.0; default parameters) was used to annotate and name predicted proteins. Results from BLASTP (version 2.2.28) of predicted *Stylonychia* proteins versus the National Center for Biotechnology Information nonredundant (nr) database (retrieved on July 29, 2013), with an *e* value threshold of 1e-3 and max_target_seqs=20, and InterProScan version 4.8 (Quevillon et al. 2005) (run in default mode) were the input for Blast2GO. Supplementary data file S2 (stylo_asm1.fixed.go_annotations.txt), Supplementary Material online, contains the final annotations from this pipeline.

### Assessment of Genome Completeness

We used three methods to assess the completeness of the draft *Stylonychia* macronuclear genome assembly: 1) The percentage of reads mapping to the assembly, 2) the percentage of conserved core eukaryotic genes (CEGs) (Parra et al. 2007) with *Stylonychia* homologs, and 3) whether a complete set of tRNA genes was predicted. All these measures indicate that the draft *Stylonychia* macronuclear genome assembly is essentially complete.

Individual read libraries were mapped to the MAC assemblies with LAST (Frith et al. 2010; Kielbasa et al. 2011) (lastal -r6 -q18 -a21 -b9 -e180) to estimate raw read coverage. Reads containing telomeric sequences were separately mapped to the assemblies using LAST, to estimate telomeric read coverage. Output MAF alignment files were converted into a SAM files with maf-convert.py from the LAST package. Mapped reads with ≥90% identical matches, covering ≥70% of the read length were counted. Almost 98% of the nontelomeric Illumina reads in our small fragment library and 92% of telomeric reads match our draft assembly (table 2). The reduced fraction of matching telomeric reads may indicate that we have missed a small fraction of alternative nanochromosome ends, but as nanochromosome ends were typically found in nongenic regions in *Oxytricha* (Swart et al. 2013) and only approximately 2% of all the reads do not map to the *Stylonychia* MAC genome assembly (table 2), we expect only a minor loss of sequence information.

For the CEG analysis, protein sequences from *Stylonychia* were BLASTed against the 248 CEGs (Parra et al. 2007). Matches from BLASTP with *e* values lower than 1e-10 and a sequence coverage ≥70% of the CEG sequence were counted as a match. Of the 248 accepted CEGs, 234 proteins predicted for *Stylonychia* were likely homologs (based on BLASTP matches; see Materials and Methods for the homology criteria). Ten of the fourteen remaining CEGs also appear to be absent in the *Oxytricha* MAC genome based on the CEGMA criteria (Parra et al. 2007) (see supplementary table S4, Supplementary Material online, for the missing BLASTP matches), but can be found in both *Stylonychia* and *Oxytricha* either by less restrictive BLASTP matches, TBLASTN matches or by using HMMER3 domain searches. After accounting for these issues, in *Stylonychia* only the MAD2 spindle assembly checkpoint protein (KOG3285) is missing from the superset of 245 CEGs from *Oxytricha*, *Paramecium*, and *Tetrahymena*. This protein is also missing from *Oxytricha* (Swart et al. 2013). Thus, the macronuclear genomes of both *Stylonychia* and *Oxytricha* encode 99.6% of the ciliate-specific CEGS.

To assess he completeness of the *Stylonychia* tRNA complement, tRNAscan-SE (version 1.3.1, run in default mode) (Lowe and Eddy 1997) was initially used to predict the tRNAs encoding for the standard 20 amino acids. To determine which tRNAs were unique, tRNA sequences were extracted and aligned with MAFFT (Katoh et al. 2002; Katoh and Standley 2013) (default parameters), followed by inspection of the subsequent alignments by eye. Selenocysteine tRNAs in *Stylonychia* and *Oxytricha* were predicted using Infernal 1.1rc4 (Nawrocki et al. 2009; Nawrocki and Eddy 2013) using the Rfam 11.0 (Griffiths-Jones et al. 2003; Burge et al. 2013) model for this tRNA. *Stylonychia*’s MAC genome encodes a comprehensive set of tRNAs for all the 20 standard amino acids and for selenocysteine.

### Ortholog Prediction

Protein sequences from both *Oxytricha* and *Stylonychia* were first clustered independently using cd-hit (v4.5.4) (Li et al. 2001; Li and Godzik 2006) with a protein clustering identity threshold of greater than 95% to merge alleles (21,490 clusters in *Oxytricha* and 20,968 clusters in *Stylonychia*). We then performed BLASTP searches of the representative, clustered protein sequences from cd-hit and selected the reciprocal best hits (7,374) using a custom Python script.

### Estimation of Nanochromosome Copy Number

To analyze relative nanochromosome copy numbers, sequencing reads from the four libraries were mapped to the Terminator 2.0 assembly using SHRiMP (version 2.2.3, run in default mode; Rumble et al. 2009; David et al. 2011). For most single-gene nanochromosomes, library fragment size does not seem to have a major effect on estimation of relative copy number (supplementary fig. S2, Supplementary Material online), and so we based our copy number estimates for the final SPAdes assembly on library 24. For each contig, mapped reads were counted and normalized by contig length (mapped reads per base).

### Determination of Alternative Fragmentation of Nanochromosomes

**Fig. 2.—:**
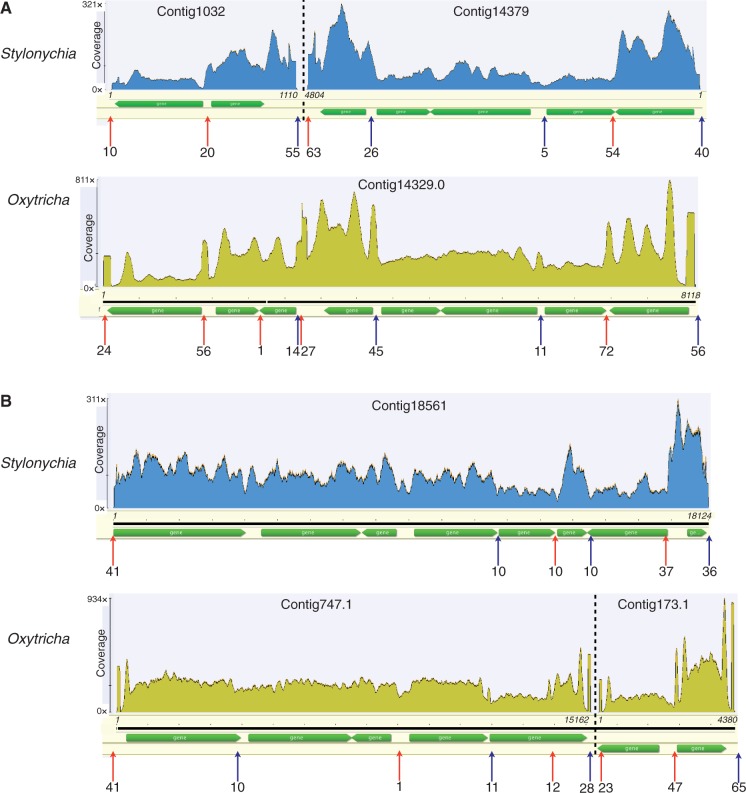
Synteny of *Stylonychia* and *Oxytricha* multigene nanochromosomes. Fold coverage of reads is indicated for total reads mapped with bwa to nanochromosomes from *Stylonychia* (library 24) and for the *Oxytricha* MAC genome Illumina library (Swart et al. 2013). *Oxytricha* has prominent doublet peaks corresponding to telomeric end coverage biases of the PE reads (this phenomenon is due to the larger fragment sizes of the *Oxytricha* Illumina library). Alternative fragmentation sites are indicated by upward pointing arrows with the number of reads corresponding to the approximate fragmentation site below. Coordinates (italicized numbers) of the contigs (in bp) are given relative to the current assembly.

To determine alternative fragmentation, we first selected all read pairs possessing a read starting with the most common telomeric repeat “CCCCAAAACCCCAAAACCCC.” The telomeric repeat was then stripped before mapping the read pairs with bwa (parameters: -n 0). For the individual nanochromosomes in figure 2 we inspected the locations of the telomere-stripped reads and determined the most frequent location of the mapped stripped ends, and the number of reads in close proximity (∼100-bp window) for this location by eye.

### Histone Phylogenies

*Saccharomyces cerevisiae* histone variants were obtained from UniProt (the histone variant accession numbers are: H3—UniProt:P61830; CenH3—UniProt:P36012; H2A.1—UniProt:P04911; H2A.2—UniProt:P04912; H2A.Z—UniProt:Q12692; H2B.1—UniProt:P02293; and H2B.2—UniProt:P02294). *Tetrahymena thermophila* histone variants were obtained from the *Tetrahymena* Genome Database (TGD) (Stover et al. 2006) (accession numbers: H3.1—TGD:TTHERM_00189180; H3.3—TGD:TTHERM_00016170; H3.4—TGD:TTHERM_00016200; CenH3—TGD:TTHERM_00146340; H2A.1—TGD:TTHERM_00316500; H2A.V—TGD:TTHERM_00143660; H2A.X—TGD:TTHERM_00790790; H2A.Y—TGD:TTHERM_01079200; H2B.1—TGD:TTHERM_00633360; and H2B.2—TGD:TTHERM_00283180). Alignments for each type of histone were generated using Geneious’s (Kearse et al. 2012) MAFFT (Katoh et al. 2002; Katoh and Standley 2013) (v7.017 default parameters) plugin, after which conserved blocks of amino acids (minus N- and C-terminal extensions for some of the histone variants) were manually selected. PhyML (Guindon et al. 2009) (LG substitution model; invariable site proportion = 0; four substitution categories; estimated gamma distribution parameter; optimization of topology and branch length; topology search by nearest neighbor interchanges) was used to generate 100 bootstrap replicates for each phylogeny.

### *Stylonychia* Macronuclear Genome Database

A genome-centric model organism database containing a GBrowse 2 genome browser (Stein et al. 2002), BLAST (Altschul et al. 1997) server, and database of gene function annotations has been established to aid research on *S. lemnae*. StyloDB (stylo.ciliate.org) was modeled after other ciliate.org websites, including those for *Tetrahymena* (Stover et al. 2006) and *Oxytricha* (Swart et al. 2013), and utilizes the same underlying architecture and programming as these projects. StyloDB features a public curation interface that allows members of the research community to edit annotations for each gene, including gene names, Gene Ontology annotations, and published references. Genome, predicted gene, and protein sequence files can also be accessed from the StyloDB website.

## Results and Discussion

### Choice of an Optimal DNA Fragment Size for Assembling Highly Fragmented Macronuclear Genomes

Currently no genome assemblers are specifically designed to cater to the unique properties of the highly fragmented stichotrich macronuclear genomes, that is, high levels of heterozygosity, nanochromosome copy number variation, alternative nanochromosome fragmentation, and variability of the telomere addition site. Nevertheless, we have produced respectable assemblies after extensive exploration of different assemblers and assembly parameters. As with the *Oxytricha* MAC genome assembly, we have not sought to resolve nanochromosome haplotypes for *Stylonychia* during genome assembly, which is a complicated problem in genomes with relatively high levels of heterozygosity (Small et al. 2007; Swart et al. 2013) (though, as we show in the next section, this is mitigated by inbreeding of *Stylonychia*).

As multiple Illumina libraries with different DNA fragment sizes were generated for this genome project (supplementary fig. S3, Supplementary Material online; libraries 24–27), we were able to evaluate how fragment size affects the quality of the assembly (table 1). We sought to maximize both the read coverage of the assembly and the number of assembled nanochromosomes, while minimizing the number of contigs. Based on these criteria, we found that the best assemblies were produced from the library with the tightest fragment size distribution and small fragments (library 24; mean outer distance of 163 bp; table 1). By virtue of the size selection procedure employed after DNA fragmentation, Illumina PE libraries with longer fragment lengths tend to have a region with low or no sequence coverage at the ends of nanochromosomes (as noted in *Oxytricha* [Swart et al. 2013], and can also be seen in fig. 2). Together with variation in the precise site of telomere addition site (Swart et al. 2013), this low coverage region may be responsible for the failure to link nanochromosome ends to many contigs. This low coverage region becomes more problematic as the library fragment size increases, for example, the fraction of telomere-bearing reads (from library 24) matching the assembly of our two larger fragment libraries, 25 and 27, is 59.5% and 62.5%, in contrast to 92.2% for the small fragment library 24 (table 1).

Combining different libraries typically did not improve the assemblies compared with the assembly of just library 24 (genome assembly quality has previously been shown to saturate and even worsen as sequencing depth increases [Magoc et al. 2013]). Using all the libraries produced a bloated assembly with only approximately 9% of the contigs possessing two telomeres (supplementary table S5, Supplementary Material online). Even though the best SPAdes assembly combination (libraries 24 and 25) produced a comparable number of complete nanochromosomes to our library 24 assembly (supplementary table S6, Supplementary Material online), it produced approximately 11,000 extra contigs. Consequently, we based our final assembly exclusively on library 24.

Based on our analyses of *Stylonychia* MAC genome assemblies, we propose using PE Illumina libraries created from short DNA fragments with the SPAdes genome assembler as a cost-effective strategy to produce well-assembled, high complexity fragmented genomes, including the macronuclear genomes of spirotrichs such as *Euplotes* (Vinogradov et al. 2012) and phyllopharyngean ciliates, such as *Chilodonella*. We also suggest the use of small fragment libraries for genome assemblies in general if the goal is to obtain the ends of chromosomes, as larger fragment sizes prevent assembly of these ends. As SPAdes was not designed for diploid genome assembly, it will still be necessary to use additional sequencing strategies for haplotype resolution in future.

### Selection of a Reference Genome Assembly

After testing multiple genome assemblers (supplementary table S1, Supplementary Material online) we found two strategies generated our “best” *Stylonychia* macronuclear genome assemblies: 1) A combination of the IDBA-UD assembler (Peng et al. 2012) and a custom extension/assembly approach (Terminator 2.0), and 2) the SPAdes genome assembler (Bankevich et al. 2012) with additional postprocessing to remove tiny, redundant contigs, followed by merging with CAP3 (Huang and Madan 1999). In our first strategy, we used an iterative procedure (Terminator 2.0) to merge and extend incomplete nanochromosomes after assembling Illumina reads with IDBA-UD (see Materials and Methods). The completeness of the two assemblies, as assessed by the total number of nanochromosomes and percentage of mapping reads, is quite similar (table 2). As it was the simpler and less computationally intensive of our two best assembly strategies, we chose the postprocessed SPAdes assembly for our reference draft assembly.

Although we were testing different genome assemblers and assembly parameters, we examined the assemblies of the most highly amplified nanochromosome and the longest nanochromosome, because they present challenging cases for the assemblers and were often not completely assembled. With both IDBA-UD/Terminator 2.0 and SPAdes we completely assembled the highest copy number nanochromosome in *Stylonychia*, encoding the large rRNA subunit (7,455 bp, including telomeres). Both IDBA-UD/Terminator 2.0 and SPAdes also completely assembled the longest *Stylonychia* nanochromosome (65,401 bp). This nanochromosome is a single gene nanochromosome which is orthologous (best reciprocal match) to the longest *Oxytricha* nanochromosome (66,022 bp; encoding the Jotin protein [Swart et al. 2013]). It should be noted that even with just single-end reads we obtained a relatively complete assembly using SPAdes (14,619 two-telomere contigs, and the complete 65.4 kb Jotin contig; table 2).

The total number of *Stylonychia* nanochromosomes (16,059) in our reference assembly is similar to that of *Oxytricha*, but the total number of contigs and assembly size is slightly smaller (∼20,000 vs. ∼22,500 contigs, and ∼50 vs. ∼67 Mb; table 2). The smaller *Stylonychia* MAC genome size is roughly consistent with sequence complexity estimates (47 Mb for *Stylonychia* vs. 55 Mb for *Oxytricha*) (Prescott 1994). Two factors are likely the main reasons for the difference in size of these assemblies: 1) The *Oxytricha* MAC genome contains some redundancy due to the use of two strains and a complex assembly strategy (Swart et al. 2013), and 2) there are lower levels of heterozygosity in the *Stylonychia* MAC genome than the *Oxytricha* MAC genome (supplementary fig. S4, Supplementary Material online). Assembled *Stylonychia* nanochromosomes are somewhat shorter on average than *Oxytricha* (mean length 2,760 bp compared with mean length 2,982 bp), which may reflect the use of Sanger reads in the *Oxytricha* assembly, and also the successive greedy CAP3 meta-assemblies, which will tend to merge nanochromosome isoforms arising from alternative fragmentation. Overall, we observe a significant improvement in the proportion of complete nanochromosomes (81% of contigs with two telomeres) in the draft *Stylonychia* macronuclear assembly compared with the draft *Oxytricha* assembly (71% of contigs with two telomeres).

### Synteny and Alternative Fragmentation

Although the taxonomic classification of stichotrichous ciliates including *S*. *lemnae* and *O*. *trifallax* has been in a state of flux (Schmidt et al. 2007; Zoller et al. 2012), at the sequence level these species are quite similar. For example, for a small set of *S. lemnae* and *O. trifallax* protein-coding genes, the 4-fold synonymous substitutions were approximately 0.4 substitutions/site (Jung et al. 2011). We decided to examine orthologous-predicted genes of *Stylonychia* and *Oxytricha* to assess how much conservation of synteny exists between nanochromosomes in the two species. We began with nanochromosomes encoding the most genes in *Oxytricha* and *Stylonychia* (eight genes encoded by two different nanochromosomes in both cases; fig. 2), as this gives us the longest regions to observe potential stretches of synteny. In *Oxytricha*, one 8-gene nanochromosome (eight; OxyDB:Contig14329.0; GenBank: AMCR01001519) is also the most extremely alternatively fragmented (producing at least 14 distinct nanochromosome isoforms) (Swart et al. 2013). The entire length of this *Oxytricha* nanochromosome aligns to two *Stylonychia* nanochromosomes (StyloDB:Contig14379 and StyloDB:Contig1032; fig. 2*A*; BLAST best-reciprocal hits to the *Oxytricha* nanochromosome). No read pairs link these contigs, even in our larger insert library (library 25). These two *Stylonychia* contigs encode five genes and two genes, respectively (as judged by BLASTX, in the latter AUGUSTUS failed to predict a gene in the region corresponding to *Oxytricha*’s gene OxyDB:Contig14329.0.g33). Colinearity between the entire *Stylonychia* and *Oxytricha* nanochromosome genes is also evident between the other *Oxytricha* eight-gene nanochromosome, OxyDB:Contig13261.0, and *Stylonychia* StyloDB:Contig909 (these two contigs align end-to-end and are 61% identical using Geneious’s [Kearse et al. 2012] Needleman–Wunsch alignment plugin with default parameters). The *Stylonychia* nanochromosome with eight predicted genes (StyloDB:Contig18561; fig. 2*B*) is syntentic with two *Oxytricha* nanochromosomes (OxyDB:Contig747.1 and OxyDB:Contig737.1, which also lack any reads supporting their linkage). Therefore, at smaller genomic scales (<20 kb), there appears to be a considerable amount of synteny between multigene nanochromosomes of *Stylonychia* and *Oxytricha*.

To explore synteny more generally, we searched for synteny among two-gene contigs using BLASTP of predicted proteins. *Stylonychia* two-gene contigs were counted as potentially syntenic with *Oxytricha* two-gene contigs if both the *Stylonychia* proteins separately matched (*e* value < 1e-10) two proteins on a *Oxytricha* contig. Using these criteria, 43% of two-gene contigs (from 2,364 two-gene contigs) in *Stylonychia* appear to be syntenic with those in *Oxytricha*. Given the conservation of synteny between the nanochromosomes of *Stylonychia* and *Oxytricha*, we desired to know how well alternative fragmentation sites are conserved between these two species. The alternative fragmentation sites for the two multigene nanochromosomes in figure 2 are usually conserved, but in both cases a site that is an alternative fragmentation site in one of the species appears to be a normal chromosome breakage site in the other species, or too weakly fragmented to be detected. More generally we found that approximately 66% of *Stylonychia*’s syntenic two-gene contigs showed alternative fragmentation (supported by at least one internally mapping telomeric read) in both species if alternative fragmentation was found in either species.

### Conservation of Relative Nanochromosome Copy Number between *Stylonychia* and *Oxytricha*

**Fig. 3.—:**
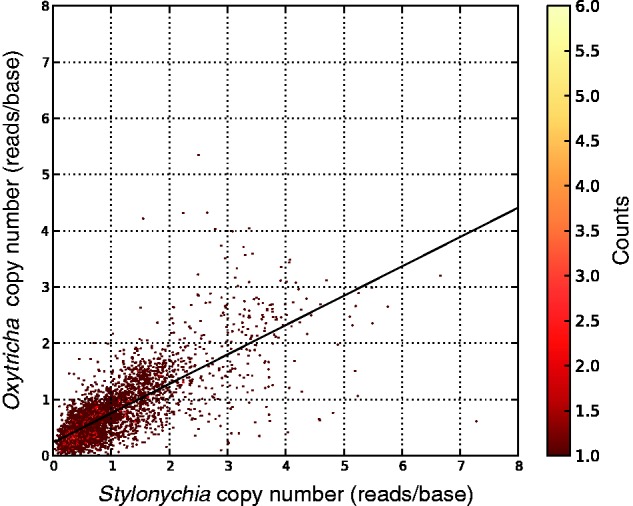
Conservation of nanochromosome copy number and length. Nanochromosome copy number was determined for nanochromosomes encoding orthologous proteins in the Terminator 2.0 assembly (see Materials and Methods). As the *Oxytricha* Illumina library is slightly smaller than that of *Stylonychia* library 24, we multiplied the *Oxytricha* reads/bp value by 1.242 (total mapped *Stylonychia* reads/total *Oxytricha* mapped reads) to normalize the library sizes.

Previously a survey of 11 orthologous nanochromosomes in *S. lemnae* and *O. trifallax* showed that the relative copy number of these nanochromosomes is similar (Xu et al. 2012). Although we were examining alternative nanochromosome fragmentation, we noticed that the patterns of sequence coverage of the different nanochromosome isoforms from *Stylonychia* and *Oxytricha* are similar (fig. 2). To examine the general relationship between the copy number of *Stylonychia* and *Oxytricha* nanochromosomes in a straightforward manner, we compared the base coverage of putative orthologous single-gene nanochromosomes (see Materials and Methods). A strong correlation between the nanochromosome copy numbers (Pearson’s *r* = 0.77) of these two species can be seen in fig. 3.

Studies in both *Oxytricha* and *Stylonychia* suggest that copy number may be epigenetically inherited across generations (Heyse et al. 2010; Nowacki et al. 2010). In one study, injection of sRNAs reduced nanochromosome copy number, and was proposed to be a consequence of degradation of putative copy number determining RNA templates by RNA interference (Nowacki et al. 2010). In the other study injection of ss- or dsRNA templates led to an increase in nanochromosome copy number, and copy number of individual nanochromosomes was also stably inherited for 100 asexual generations (Heyse et al. 2010), as predicted by stochastic models of nanochromosome segregation (Duerr et al. 2004).

Consistent with the stochastic segregation model, the copy number of specific nanochromosomes occasionally becomes highly overamplified (up to ∼100× the normal copy number) (Steinbrück 1983; Harper et al. 1991). As an argument for genetic control of copy number determination, in crosses of *Stylonychia* strains with such overamplified nanochromosomes their progeny initially showed no overamplification of these nanochromosomes, but after approximately 12 months some of the newly created strains showed overamplification of some of the nanochromosomes, whereas other strains showed no overamplification (Steinbrück 1983). If nanochromosome copy number was solely epigenetically controlled, there would be no way for such large copy number fluctutations to be brought back to normal levels during sexual reproduction. Moreover, epimutation rates appear to be orders of magnitude higher than typical eukaryotic genetic mutation rates (Jablonka and Raz 2009; Schmitz et al. 2011), and so, over time, we expect that epigenetic copy number control would lead to wide variation in nanochromosome copy number. Given these problems with epigenetic copy number control, we concur with Steinbrück (1983) that the establishment of nanochromosome copy number during new macronuclear development has an important genetic component.

### *Stylonychia*’s MAC Genome Encodes DDE_3 Transposase Genes

We briefly examined the protein domain complements of *Stylonychia* and *Oxytricha* to see whether we could identify any interesting species-specific proteins, but in general, as these two ciliates are relatively closely related, their protein domain complements are also quite similar (See supplementary data file S1, Supplementary Material online: “Known protein domains are conserved between *Stylonychia* and *Oxytricha*”). We therefore only consider a few classes of proteins of special interest in the remainder of this article.

In ciliates domesticated transposases involved in DNA elimination and genome reorganization may either be expressed from the micronuclear genome, as for the *Oxytricha* micronuclear genome-limited TBE (telomere-bearing element [Herrick et al. 1985]) transposases (Nowacki et al. 2009), or be expressed from the macronuclear genome like the PiggyBac-related transposases of *Paramecium* (Baudry et al. 2009) and *Tetrahymena* (Cheng et al. 2010). In addition to the TBE transposases, *Oxytricha* also has two families of transposases: MULE (Pfam:PF10551) transposases and ISXO2-like (Pfam:PF12762) transposases (Swart et al. 2013). Both of these nanochromosome-encoded transposase families were also found among the present predicted *Stylonychia* proteins (see supplementary data file S1, Supplementary Material online). Interestingly, we found three Pfam DDE_3 transposase domain matching proteins, encoded on telomere bearing contigs (StyloDB: Contig3970.g4257, Contig6146.g6579 and Contig13700.g14613). The DDE_3 domain is characteristic of *Oxytricha*’s micronuclear-encoded TBE transposases (two predicted proteins in the current *Oxytricha* MAC genome assembly [OxyDB:Contig5254.0.g70 and Contig2394.0.g82] appear to be TBE transposases encoded on telomereless contigs [potentially MIC genome contaminants] [Swart et al. 2013]). When we used the *Stylonchia* nanochromosome-encoded DDE_3 proteins to query GenBank’s nr database with BLASTP we found no matches to *Oxytricha* TBE transposases, suggesting that the transposases in *Oxytricha* may only be distantly related to the nanochromosome-encoded DDE_3 *Stylonychia* proteins. It will be of interest to assess whether *Stylonychia*’s nanochromosome-encoded DDE_3 transposases are developmentally expressed like most known *Oxytricha* transposase genes, and whether they are involved in genome rearrangements like the TBE transposases.

### Two New Telomere End-Binding Beta Proteins

In the *Oxytricha/Stylonychia* MAC, the major telomere-binding protein complex is comprised a dimer of telomere-binding protein alpha (TeBPα) and telomere-binding protein beta (TeBPβ) (Lipps et al. 1982; Gottschling and Zakian 1986). While examining the predicted *Oxytricha* proteome we found five additional TeBPα proteins and two additional TeBPβ proteins (and hence we now refer to the original telomere-binding proteins as TeBPα1 and TeBPβ1). Since we performed this search, a new domain, corresponding to the human telomere protein, TPP1, has been added to the Pfam database (version 27). We found matches to the TPP1 domain (PF10341) in both *Stylonychia* and *Oxytricha* (two pairs of orthologous [best-reciprocal hits] proteins each). The TPP1 domain of one of these proteins (StyloDB:Contig8366.g8920, OxyDB:Contig1486.1) overlaps its Pfam TeBPβ domain (Pfam:PF07404). This is consistent with the structural homology found between human TPP1 and *O. nova* TeBPβ1 (Xin et al. 2007), and suggests that these Pfam domains could be unified.

Counting proteins with either the TPP1 domain or TeBPβ domain, there are five distinct TeBPβ proteins in *Stylonychia*. The best reciprocal hit to one of these *Stylonychia* proteins (StyloDB:Contig2512.g2701) in *Oxytricha* (OxyDB:Contig19388.0.g78) does not have a detectable TPP1 domain or TeBPβ domain (at a threshold *e* value < 1.0). These proteins are both relatively long (836 and 1,092 aa), and, excluding an approximately 172-aa N-terminal extension in *Oxytricha*, their pairwise alignment is approximately 34% identical (alignments by MAFFT [Katoh et al. 2002; Katoh and Standley 2013] version 7.017; default parameters).

Our inspection of the domain architectures of 102 TPP1-domain containing proteins from the Pfam website (Punta et al. 2012) indicates that this domain is usually located in the N-terminal portion of the protein and often has a long C-terminal region (>200 aa) with no predicted domains, as is the case for all the *Stylonychia/Oxytricha* TeBPβ proteins. Similarly, aside from TeBPα1, which has multiple POT1 domains (Pfam:PF02765; known as “Telo_bind” in Pfam version 26), other *Stylonychia*/*Oxytricha* TeBPα proteins only have an N-terminal POT1 domain. This suggests that, relative to the remainder of these proteins, their N-terminal regions are subject to stronger purifying selection.

The restricted distribution of the TPP1 domain among eukaryotes is striking: Of the 151 protein sequences with detectable TPP1 Pfam domains in UniProt (release 2013_12), only three, including two *Oxytricha* matches, were not found in opisthokonts (including animals and fungi). The only other nonopisthokont match we found was to an *Acanthamoeba castellanii* protein (UniProt:L8H2E8_ACACA). The TeBPβ Pfam domain only has matches to proteins from *Oxytricha* and *Stylonychia* species. Unless other eukaryotes possess very divergent, and consequently as yet undetected homologs of TPP1, this suggests the absence of TPP1 in ancestral eukaryotes, and the possibility that this protein may have been acquired horizontally by the common ancestor of some ciliates. Although there is a proposed “functional homolog” of TPP1 in *Tetrahymena* (corresponding to the N-terminal of the protein prediction TGD:TTHERM_00523050) (Linger et al. 2011), we cannot detect either the TPP1 domain or TeBPβ domain in this protein. No TPP1 homolog has been proposed or detected in *Paramecium*. We previously found a single protein in *Paramecium* with a POT1 domain (Swart et al. 2013) and *Tetrahymena* is known to have two POT1 proteins (Jacob et al. 2007), so these ciliates do appear to have homologs of TeBPα.

### *Stylonychia* Has Two Linker Histone Proteins

As chromatin biology is an active area of research in *Stylonychia* and other ciliates (e.g., Bulic et al. 2013; Gao et al. 2013; Shieh and Chalker 2013; Vogt and Mochizuki 2013; Forcob et al. 2014) we were interested in characterizing the complete diversity of core histones, which is described in the next results section. First we searched for linker histones, as, despite extensive characterization of these histone proteins in *T. thermophila* (Gorovsky et al. 1974; Gorovsky and Keevert 1975; Allis et al. 1984; Wu et al. 1986, 1994; Hayashi et al. 1987; Shen and Gorovsky 1996; Dou et al. 1999; Dou and Gorovsky 2000, 2002), and identification and sequencing of a histone H1 gene in *Euplotes eurystomus* macronuclei (Herrmann et al. 1987; Hauser et al. 1993), no linker histone sequences have been reported in other ciliates. *Tetrahymena* has a single gene encoding its macronuclear histone H1 (Wu et al. 1986), and another gene (MLH) encoding a set of four linker histone proteins as a polyprotein (Allis et al. 1984; Wu et al. 1994). Two of the protein forms generated from the MLH gene have an HMG box domain (Wu et al. 1994) (matching the Pfam domain HMG_box). No sequence similarity was observed between the histone H1 gene of *Euplotes eurystomus* and the globular histone H1 domain from other eukaryotes (Hauser et al. 1993).

From the two-dimensional SDS-PAGE analyses of *O. nova* and *Stylonychia*, it was inferred that histone H1 was missing at the expected location (compared with *Tetrahymena* and Chicken) for acid-extracted proteins from macronuclei (Butler et al. 1984). However, low mobility, 20–30 kDa proteins were noted in the *Oxytricha* protein extracts, and it was suggested that if histone H1 proteins are present in *Oxytricha* or *Stylonychia*, they might have major biochemical differences from those in animals (Butler et al. 1984). Two putative histone H1 protein bands were identified in an earlier study of *Oxytricha* histone extracts and were lysine rich compared with the other histones (Caplan 1977). Putative H1 histones were absent from micronuclear extracts in this study, but were identified in the micronuclear extracts of *Stylonychia* (Schlegel et al. 1990). We therefore desired to know whether any candidate histone H1 genes could be found in *Stylonychia*.

Among our HMMER3 domain annotations we noticed a single convincing match to the Pfam histone H1 domain (linker_histone - Pfam:PF00538) in *Stylonychia* (StyloDB:Contig14654.g15612, *e* value = 7e-8), but no such match in *Oxytricha*. By BLASTP searches, we found protein homologs of this histone (H1.1) in *Oxytricha*: Two shorter, identical proteins (OxyDB:Contig14754.0.0.g58 and OxyDB:Contig10099.0.1.g76; 220 aa; *e* value = 7e-28) and an additional longer protein (OxyDB:Contig20723.0.g17; 501 aa; *e* value = 2e-10)*.* The best reciprocal hit to the longer *Oxytricha* protein in *Stylonychia* is a 356 aa protein (StyloDB:Contig2637.g2828; histone H1.2). The orthologs of histone H1.1 are approximately 51.8% identical, and the orthologs of histone H1.2 are approximately 29.% identical, excluding unmatched C-terminal regions. Multiple sequence alignment with MAFFT (Katoh et al. 2002; Katoh and Standley 2013) revealed that the region corresponding to the first approximately 49 aa acids of the histone domain match in *Stylonychia* H1.1 aligns without gaps and is conserved among all the *Stylonychia* and *Oxytricha* histone H1 variants (41% of the sites are identical and the mean pairwise identity between the pairs is 66%). As is the norm for eukaryotic histone H1, and consistent with the lysine richness of putative histone H1 proteins from *Oxytricha* (Caplan 1977), these new histone H1 variants are lysine rich (∼20% lysine: Approximately double the lysine content of other *Stylonychia/Oxytricha* histones).

### *Stylonychia*’s Cornucopia of Core Histone Variants

Following the discovery of a large number of histone H3 variants in *Stylonychia* (Bernhard 1999) their localization patterns and functions have begun to be teased apart (Postberg et al. 2008; Forcob et al. 2014). An unusually large histone H3 protein (“protein X”; molecular weight 21,000) previously observed in *Stylonychia* micronuclear histone extracts (Schlegel et al. 1990) has recently been identified as a divergent histone H3 variant, H3.8, and appears to be replaced in the developing new MAC during macronuclear development, including by the H3.7 variant (Forcob et al. 2014). No histone H3 variant of a typical, smaller eukaryotic histone H3 size was found in micronuclear histone extracts (Schlegel et al. 1990). Differences in migration of histone H2A and H2B proteins were noted between the micronuclear and macronuclear *Stylonychia* histone extracts and were suggested to be due to modifications of these proteins (Schlegel et al. 1990) as no variants of these histones were known.

As relatively little is known about histone H2A and H2B variants in *Stylonychia* we decided to examine the diversity of these variants among our gene predictions, and at the same time to check whether any other histone H3 or H4 variants were previously missed. We searched for proteins in *Stylonychia* possessing the Pfam core histone domain using HMMER3 (Eddy 2013) (Pfam:PF00125; *e* value < 1e-6). In total we found 21 *Stylonychia* histones with this domain, corresponding to 19 distinct histone variants: One histone H4 protein, nine histone H3 proteins, six histone H2A proteins, and four histone H2B proteins. The diversity of *Stylonychia* histone variants is almost double that of *T. thermophila* (12; proteins from ciliate.org, November 26, 2013, possessing the Pfam core histone domain). Likely as a consequence of multiple whole-genome duplications (Aury et al. 2006), the record holder for histone variants among ciliates is *Paramecium tetraurelia*, with 30 distinct histone variants (histone H4: 5, histone H3: 10; histone H2B: 6, and histone H2A: 9). For the purposes of comparison, we found 48 distinct human histone variants in UniProt (December 1, 2013).

There appear to be two paralogous genes encoding histone H4, as previously reported (Wefes and Lipps 1990), with identical amino acid sequences. The coding sequences of these histones are 92.7% identical in *Stylonychia* and 95.6% identical in *Oxytricha*, but both have surrounding noncoding regions that are much more divergent than typically seen between alleles. In contrast to the *Stylonychia*/*Oxytricha* histone H4 paralogs, which are invariant at the amino acid level within and between these species, and which do not appear to be particularly divergent compared with variants within other major eukaryotic groups (e.g., fungi), a number of histone H4 variants appear to have diverged substantially and evolved independently in other ciliate classes (Katz et al. 2004). We found one new histone H3, H3.9, in addition to those previously reported (Postberg et al. 2010; Forcob et al. 2014). Other than the possible independent duplications of H3.1/H3.2 variants, the same histone variants in *Stylonychia* are encoded by the *Oxytricha* MAC genome (see supplementary table S4, Supplementary Material online).

**Fig. 4.—:**
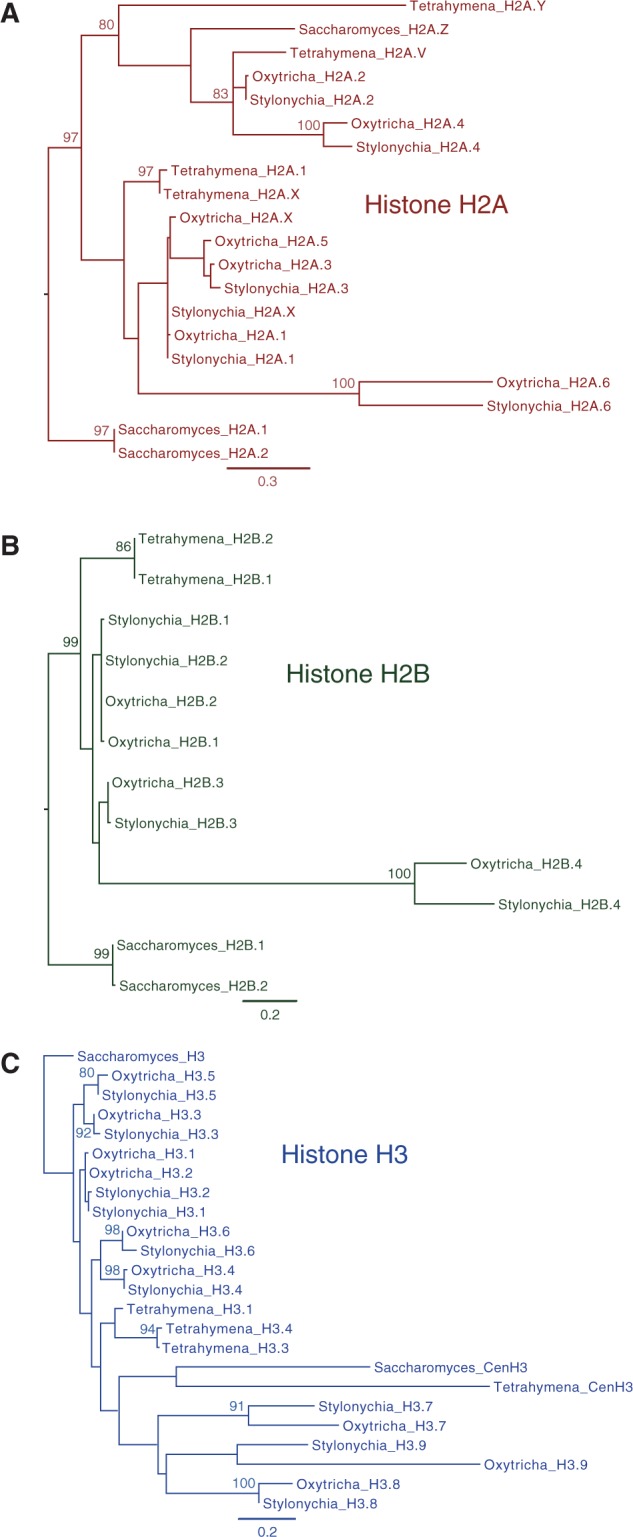
Histone variants in *Stylonchia*. Scale bars in expected substitutions per site are provided below each phylogeny and bootstrap percentages for branch points are shown when greater than 80%. Phylogenies were rooted using *Saccharomyces cerevisiae* histone variants (H2A.1/H2A.2, H2B.1/H2B.2, H3, for 4*A–C*, respectively) as outgroups. Note that in (*C*) it is possible that long-branch attraction is causing the most divergent histone H3 variants to cluster. See Materials and Methods for a list of accessions for the *Saccharomyces cerevisiae* and *Tetrahymena thermophila* histone variants.

In *Stylonychia*, we noticed five very divergent histone variants: One histone H2A, one histone H2B and three H3 histones, including the previously reported histones H3.7 and H3.8 (Forcob et al. 2014), and the new histone H3.9 (fig. 4). The divergences between the three orthologous pairs of divergent histone H3 variants (H3.7, H3.8, and H3.9) in *Stylonychia* and *Oxytricha* are much greater than between the other H3 variants (fig. 4). Pairwise MAFFT (Katoh et al. 2002; Katoh and Standley 2013) alignments of the orthologous pairs of proteins were approximately 58%, 42%, 49% and approximately 35% identical (excluding the first few amino acids which create a large gap, and starting from the first block of amino acids to the end of the protein), respectively, for divergent histone variants H2A.6, H2B.4, H3.7, and H3.9. The divergence levels among these histone variants are comparable to the divergence (48% identity) between the most highly specialized human histone variant, H2A.B (Barr body-deficient; absent from inactive X chromosomes in females), and canonical human H2A histones (Gonzalez-Romero et al. 2008), and between human centromeric histone and histone H3, for example, 47.3% identity between CENPA (UniProt:P49450) and H3F3A (UniProt:P84243). Within the H2A and H3 histone families in *Tetrahymena* there are also highly divergent histone variants, for example, HTA.V and H2A.Y are 52.7% and 47.0% identical compared with H2A.1 (fig. 4*A* and *C*). The extreme divergences of the *Stylonychia*/*Oxytricha* histone variants suggest that their functional roles may be somewhat unconventional. It has previously been suggested that the unique genome architecture may have led to elevated divergence between paralogs in ciliates with highly fragmented macronuclear genomes compared with those that are not highly fragmented (Zufall et al. 2006). However, based on the development-specific gene expression of these paralogs, together with previous demonstration of development-specific localization of the H3.7 variant (see next paragraphs), we suggest that at least for the most divergent *Stylonychia/Oxytricha* histones, functional specialization in macronuclear genome development, rather than genome architecture per se, may be the main evolutionary driving force.

**Fig. 5.—:**
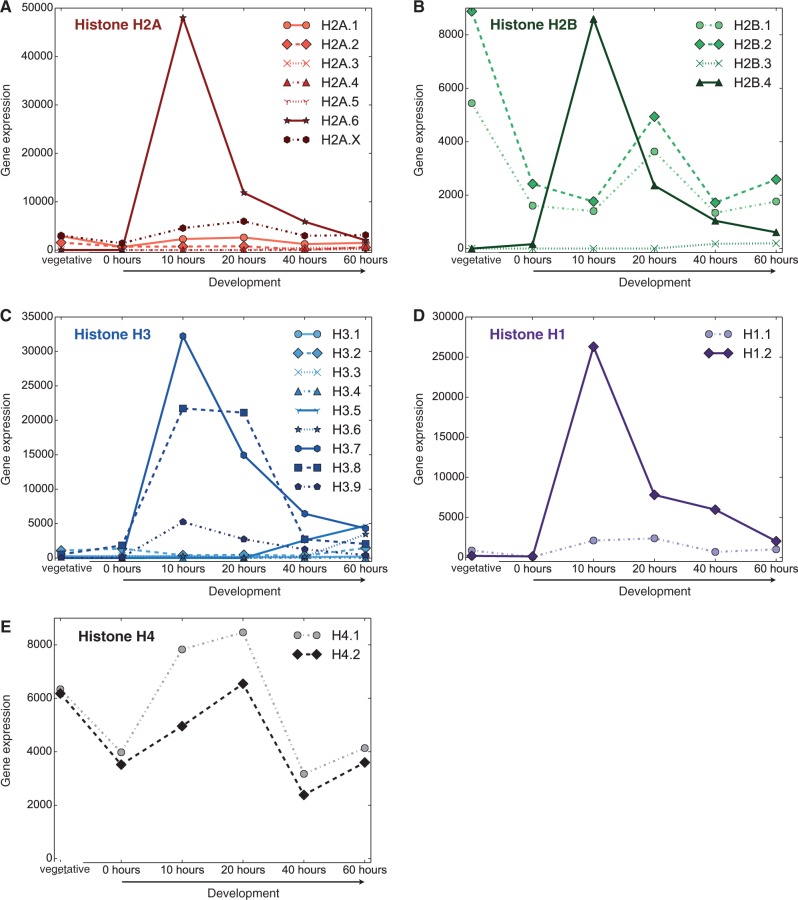
Expression of histone variants in *Oxytricha trifallax*. Gene expression values are the normalized RNA-seq counts obtained from (Swart et al. 2013) and are given in arbitrary units. “Vegetative” represents a normally fed cell culture. The developmental time course on the *x* axis starts at 0 h when cells from the complementary mating types of *O. trifallax* were mixed together (see Swart et al. 2013 for additional details about this experiment).

We were able to find mRNA sequences corresponding to each of the divergent histone variants in a cDNA library created by subtraction of vegetative cDNA from cDNA from *Stylonychia* cells 10 h postconjugation (Paschka et al. 2005). From quantitative polymerase chain reaction data *Stylonychia*’s histone H3.7 was also shown to be the most highly expressed H3 variant during the development of the new MAC, and was upregulated approximately 7 or 8 orders of magnitude compared with its vegetative expression (Forcob et al. 2014). In *Oxytricha* the orthologs of three of these divergent histones (H2B.4, H2A.6, and H3.7) appear to be highly upregulated and coexpressed, peaking early during early development before tapering off over time, and are all negligibly expressed during vegetative growth (fig. 5). The divergent histone H3.8 is highly expressed at both 10 and 20 h, and is moderately expressed during vegetative growth in *Oxytricha*. H3.9 is negligibly expressed during vegetative growth, and highly upregulated at the 10-h time point, but is expressed at lower levels than H3.7 or H3.8. The *Oxytricha* linker histone H1.2 exhibits a strikingly similar gene expression pattern to the divergent, highly expressed, development specific core histone variants, suggesting co-regulation of all these genes.

Recently, histone H3.7 was identified as a key histone in *Stylonychia*’s macronuclear genome development, specifically localizing to the developing MAC during polytene DNA formation, and disappearing after the completion of DNA elimination (Forcob et al. 2014). Silencing the histone H3.7 gene prevented further development and was usually lethal (Forcob et al. 2014). As this histone variant was shown to be enriched in macronuclear-destined DNA, it was proposed that it might be required for permissive chromatin formation (Forcob et al. 2014). The massive upregulation and coexpression of the *Oxytricha* H1.2, H2A.6, H2B.4, and H3.7 histone variants during sexual development raises the intriguing possibility of their coassembly in both *Stylonychia* and *Oxytricha*. Consequently, it will be of great interest to determine whether these histones colocalize in the developing new MAC, and particularly whether they have evolved to form a novel type of nucleosome specific for genome rearrangements.

## Supplementary Material

Supplementary data files S1 and S2 are available at *Genome Biology and Evolution* online (http://www.gbe.oxfordjournals.org/).

Supplementary Data
