# Supplementary material for: The Draft Assembly of the Radically Organized Stylonychia lemnae Macronuclear Genome
Source: Genome Biol Evol. 2014 Jun 20;6(7):1707–23. doi: 10.1093/gbe/evu139 (PMC4122937; doi:10.1093/gbe/evu139)
Supplement: Supplementary Data [file supp_6_7_1707__index.html]

The draft assembly of the radically organized Stylonychia lemnae macronuclear genome — The Draft Assembly of the Radically Organized Stylonychia lemnae Macronuclear Genome — Supplementary Data 

# The Draft Assembly of the Radically Organized *Stylonychia lemnae* Macronuclear Genome

## Supplementary Data

files

**Files in this Data Supplement:**

- Supplementary Data - zip file
