# Supplementary material for: The Draft Assembly of the Radically Organized Stylonychia lemnae Macronuclear Genome
Source: Genome Biol Evol. 2014 Jun 20;6(7):1707–23. doi: 10.1093/gbe/evu139 (PMC4122937; doi:10.1093/gbe/evu139)
Supplement: Supplementary Data [file supp_evu139_suppl_data.zip › Supplementary_data1.docx]

This file contains:

Supplementary Figures S1-S4

Supplementary Tables S1-S6

Supplementary text: "Known protein domains are conserved between *Stylonychia* and *Oxytricha*"

Supplementary figures

**Fig. S1. Terminator 2.0 meta-assembly pipeline.**

**Fig. S2. Correlation of copy number estimates from different *Stylonychia* Illumina libraries.** Axes are log_2_(reads/bp) without any normalization for library size. Copy numbers were determined for orthologous nanochromosomes from the Terminator 2.0 assembly.

**
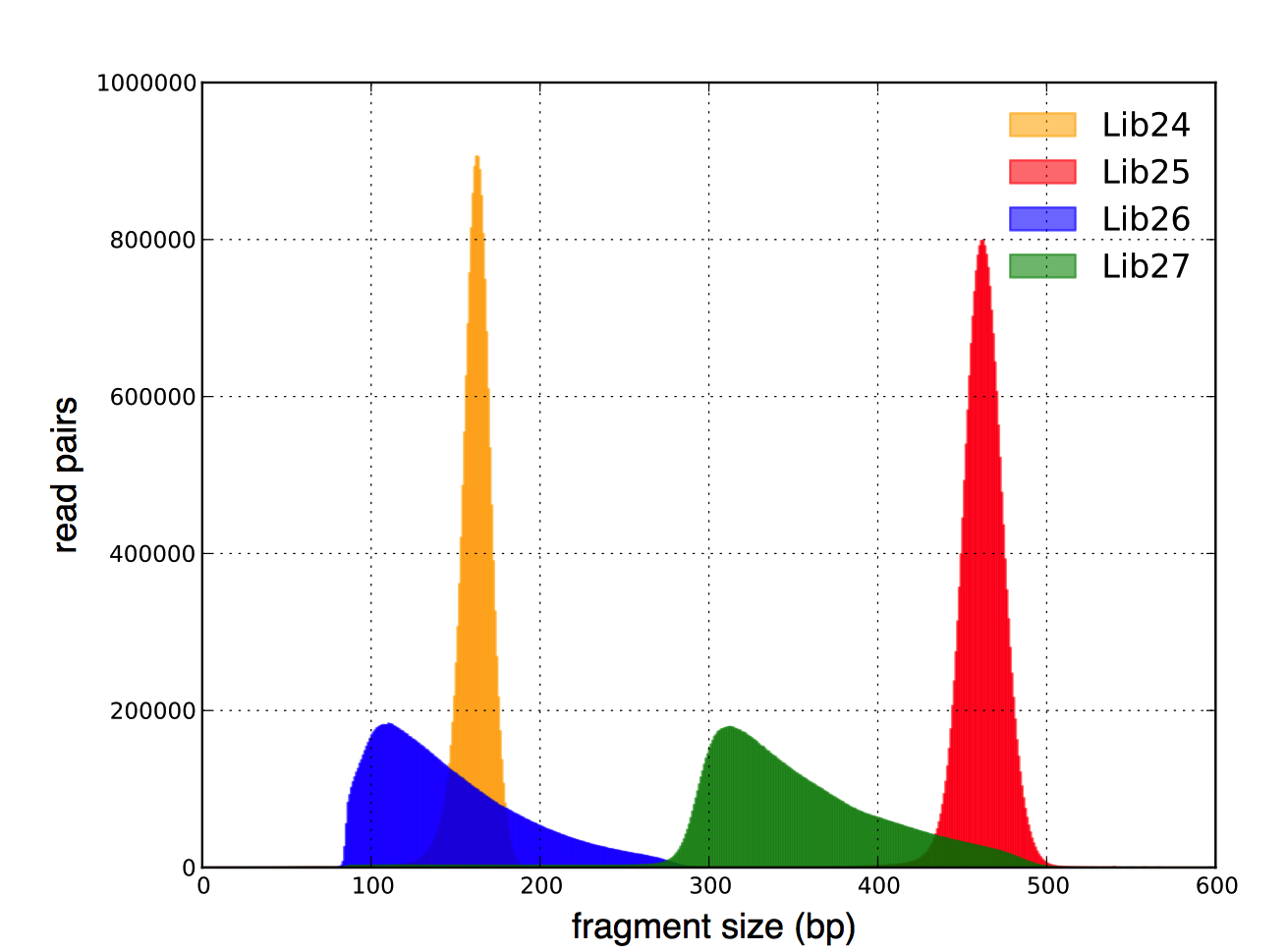
**

**Fig. S3. Fragment size distributions of *Stylonychia* MAC DNA Illumina PE libraries.** Four different libraries with read lengths of 90 bp and different insert size distributions were sequenced. Mean read pair outer distances for library 24 to 27 are: 163 bp, 463 bp, 153 bp and 361 bp, respectively. Distances were calculated by mapping reads to contigs using BWA (version 0.6.2) and a Python script slightly modified from (<https://gist.github.com/davidliwei/2323462#file-getinsertsize-py>). With the exception of library 25 (~52 million reads) the remaining three libraries are similar sizes (38-39 million reads).

**Fig. S4. Comparison of *Stylonychia* and *Oxytricha* nanochromosomal polymorphism levels of putative orthologous nanochromosomes.** SNP per base values were determined using samtools from the output of reads mapped by BWA (see Materials and Methods).

Supplementary Tables

**Table S1. Summary of genome assemblies.**

| **Assembler** | **ABySS** | **IDBA_UD** | **Meta-Velvet** | **Minia** | **Mira** | **SPA-des** | **SPA-des** | **SOAP** | **Velvet** | **Termi-nator** | **Final** |
| --- | --- | --- | --- | --- | --- | --- | --- | --- | --- | --- | --- |
| **Assembler version** | 1.3.4 | 1.0.9 | 1.2.02 | 1.5366 | 3.4.1.1 | 2.4.0 | 2.5.0 | 2.01 | 1.2.07 | 2 | N/A |
| **Assembly size (Mb)** | 58.7 | 54.9 | 57.3 | 43.1 | 51.2 | 53.5 | 52.8 | 78.5 | 48.1 | 54.7 | 50.2 |
| **Contigs (n)** | 258805 | 37366 | 182444 | 52895 | 50072 | 32021 | 29175 | 208229 | 117112 | 22758 | 19851 |
| **Telomeres (n)** | 46976 | 31973 | 29586 | 10931 | 30062 | 36163 | 34815 | 35199 | 22526 | 35961 | 34327 |
| **Mean contig length (bp)** | 227 | 1469 | 314 | 814 | 1022 | 1672 | 1811 | 377 | 411 | 2404 | 2531 |
| **Max contig length (bp)** | 22446 | 22480 | 21686 | 22449 | 65387 | 65396 | 65401 | 18861 | 19430 | 65407 | 65401 |
| **2-telomere contigs (n)** | 1634 | 9594 | 3104 | 1557 | 7776 | 15956 | 16082 | 4127 | 3864 | 16082 | 16059 |
| **1-telomere contigs (n)** | 43692 | 12691 | 23359 | 7806 | 14469 | 4181 | 2550 | 26910 | 14782 | 3683 | 2104 |
| **0-telomere contigs (n)** | 213479 | 15081 | 155981 | 43532 | 27827 | 11884 | 10543 | 177192 | 98446 | 2993 | 1688 |
| **Number of multitelomere contigs** | 16 | 87 | 19 | 11 | 40 | 61 | 76 | 32 | 16 | 105 | 79 |
| **Total PE read coverage (%)** | 91.5 | 97.6 | 92.7 | 88.9 | 97.3 | 97.9 | 98.1 | 95.6 | 92.6 | 98.2 | 98.0 |
| **Telomeric PE read coverage (%)** | 61.0 | 84.1 | 70.8 | 45.1 | 86.1 | 90.4 | 92.2 | 86.3 | 72.7 | 90.4 | 91.0 |
| **2-telomere contig percentage of all contigs** | 0.6 | 25.7 | 1.7 | 2.9 | 15.5 | 49.8 | 55.1 | 2 | 3.3 | 70.7 | 80.9 |

**Table S2. Gene predictions for complete, non-alternatively fragmented nanochromosomes from *Stylonychia*.**

| **Feature** | **Number** | **Mean (bp)** | **Min (bp)** | **Max (bp)** |
| --- | --- | --- | --- | --- |
| Nanochromosomes | 14069 | 2545 | 504 | 65401 |
| Genes | 15102 | 1920 | 146 | 64968 |
| Exons | 37150 | 728 | 3 | 49542 |
| Introns | 22048 | 89 | 29 | 467 |
| 5’ regions upstream of start codon | 12268 | 221 | 41 | 5253 |
| 3’ regions downstream of stop codon | 12268 | 184 | 31 | 7470 |

**Table S3. Gene predictions for complete, non-alternatively fragmented nanochromosomes from *Oxytricha*.**

| **Feature** | **Number** | **Mean (bp)** | **Min (bp)** | **Max (bp)** |
| --- | --- | --- | --- | --- |
| Nanochromosomes | 12479 | 3009 | 314 | 66022 |
| Genes | 13728 | 2183 | 146 | 65451 |
| Exons | 37549 | 790 | 3 | 45409 |
| Introns | 23799 | 93 | 28 | 549 |
| 5’ regions upstream of start codon | 11279 | 291 | 41 | 5400 |
| 3’ regions downstream of stop codon | 11279 | 237 | 23 | 6530 |

**Table S4. CEGs missing from BLASTP results using default CEGMA criteria.**

| **CEG name** | ***Stylonychia*** | ***Oxytricha*^(1)^** |
| --- | --- | --- |
| DNA-directed RNA polymerase, subunit RPB10 | KOG3497 |  |
| Mitochondrial F1F0-ATP synthase, subunit delta/ATP16 | KOG1758 | KOG1758 |
| 6-phosphogluconate dehydrogenase**^(2)^** | KOG2653 | KOG2653 |
| Sugar (pentulose and hexulose) kinases | KOG2531 | KOG2531 |
| Predicted snRNP core protein | KOG3448 |  |
| Glucose-6-phosphate 1-dehydrogenase**^(2)^** | KOG0563 | KOG0563 |
| OTU (ovarian tumor)-like cysteine protease |  | KOG2606 |
| 6-phosphogluconolactonase- like protein**^(2)^** | KOG3147 | KOG3147 |
| Spindle assembly checkpoint protein**^(3)^** | KOG3285 | KOG3285 |
| RNA polymerase II transcription initiation/nucleotide excision repair factor TFIIH, subunit SSL1 | KOG2807 |  |
| UDP-glucose pyrophosphorylase | KOG2638 | KOG2638 |
| Mitochondrial import inner membrane translocase, subunit TIM13 | KOG1733 | KOG1733 |
| Translation initiation factor 3, subunit g (eIF-3g) | KOG0122 | KOG0122 |
| Uncharacterized conserved protein | KOG2967 |  |
| Mitochondrial import inner membrane translocase, subunit TIM9 |  | KOG3479 |
| Predicted translation initiation factor related to eIF-2B alpha/beta/delta subunits (CIG2/IDI2) | KOG1468 | KOG1468 |
| Ubiquitin fusion-degradation protein |  | KOG1816 |
| Uncharacterized conserved protein |  | KOG3237 |
| Molecular chaperone Prefoldin, subunit 4 |  | KOG1760 |
| Small nuclear ribonucleoprotein (snRNP) SMF |  | KOG3482 |
| 60S ribosomal protein L38 |  | KOG3499 |
| **Total missing CEGs** | **14** | **17** |

(1) Hits reported missing from [13]; (2) Not found in *Tetrahymena*, *Paramecium*, *Styloncyhia* or *Oxytricha*, even with less restrictive BLAST matches or HMMER3 searches; (3) Missing in both *Stylonychia* and *Oxytricha* even with less restrictive BLAST matches or HMMER3 searches.

**Table S5. SPAdes genome assemblies for different Illumina library combinations.**

| **Library:** | **Lib24+ 25** | **Lib24+ 26** | **Lib24+ 27** | **Lib25+ 26** | **Lib25+ 27** | **Lib26+ 27** | **All libs** |
| --- | --- | --- | --- | --- | --- | --- | --- |
| Assembly size (Mb) | 58.2 | 59 | 68.7 | 57.9 | 66.8 | 61.7 | 81.5 |
| Contigs (n) | 40278 | 49414 | 82249 | 45227 | 83106 | 56472 | 133105 |
| Telomeres (n) | 37384 | 43914 | 41118 | 38672 | 26312 | 37023 | 67521 |
| N50 | 2949 | 2835 | 1742 | 2937 | 1882 | 2396 | 1563 |
| Mean contig length (bp) | 1446 | 1193 | 835 | 1280 | 804 | 1092 | 612 |
| Max contig length (bp) | 65404 | 65414 | 56757 | 65422 | 59053 | 65436 | 36319 |
| 2-telomere contigs | 16740 | 16763 | 13632 | 13915 | 5601 | 12727 | 11971 |
| Mean 2-telomere contig length (bp) | 2773 | 2757 | 2390 | 2759 | 2471 | 2700 | 2321 |
| 1-telomere contigs | 3783 | 10294 | 13746 | 10762 | 15071 | 11501 | 43512 |
| Mean 1-telomere contig length (bp) | 1154 | 452 | 997 | 875 | 1144 | 835 | 410 |
| 0-telomere contigs | 19755 | 22357 | 56871 | 20550 | 62434 | 32244 | 77622 |
| Mean 0-telomere contig length (bp) | 379 | 363 | 424 | 493 | 574 | 551 | 463 |

**Table S6. Histone variants in *Stylonychia* and *Oxytricha*.**

| **Class** | **Variant** | ***Stylonychia* gene id** | ***Oxytricha* gene id^(1)^** | **Ortholog amino acid identity** |
| --- | --- | --- | --- | --- |
| H4 | H4 | Contig13802.g14721 | Contig93.1.g73 | 100.0% |
| H4 | H4 | Contig8089.g8626 | Contig10148.0.g40 | 100.0% |
|  |  |  |  |  |
| H3 | H3.4 | Contig10759.g11510 | Contig3745.0.g52 | 99.3% |
| H3 | H3.7 | Contig610.g675 | Contig22722.0.g27 | 48.9% |
| H3 | H3.8 | Contig13116.g13984 | Contig1305.1.g89 | 84.0% |
| H3 | H3.1 | Contig14585.g15537 | Contig9358.0.g54^(2)^ | 100.0% |
| H3 | H3.2 | Contig2639.g2830 | Contig4701.0.g33^(2)^ | 100.0% |
| H3 | H3.3 | Contig14361.g15298 | Contig938.1.g137 | 96.4% |
| H3 | H3.6 | Contig14275.g15200 | Contig1081.1.g60 | 97.1% |
| H3 | H3.5 | Contig3614.g3856 | Contig12297.0.g10 | 97.1% |
| H3 | H3.9 | Contig17317.g18436 | Contig13492.0.0.g46 | ~34.7% |
|  |  |  |  |  |
| H2B | H2B.1 | Contig13656.g14566 | Contig22996.0.g96 | 90.0% |
| H2B | H2B.2 | Contig10417.g11107 | Contig678.1.g53 | 96.1% |
| H2B | H2B.4 | Contig5476.g5852 | Contig14821.0.g11 | 42.1% |
| H2B | H2B.3 | Contig13582.g14493 | Contig9113.0.g47 | 94.9% |
|  |  |  |  |  |
| H2A | H2A.6 | Contig13277.g14169 | Contig14080.0.1.g76_5 | 57.6% |
| H2A.X | H2A.X | Missing prediction (Contig7961) | Contig22612.0.g35_6 | 96.2% |
| H2A | H2A.1 | Contig2427.g2616 | Contig9772.0.g99 | 98.5% |
| H2A | H2A.2 | Contig3210.g3426 | Contig16838.0.g30 | 99.2% |
| H2A | H2A.3 | Contig2600.g2794 | Contig8958.0.g2 | 78.5% |
| H2A | H2A.4 | Contig7394.g7902 | Contig17088.0.g8 | 84.7% |
| H2A | H2A.5 | Contig2600.g2794 | Contig9320.0.g50 | 93.0% |
|  |  |  |  |  |
| H1 | H1.1 | Contig14654.g15612 | Contig10099.0.1.g76 | 50.0% |
| H1 | H1.2 | Contig2637.g2828 | Contig20723.0.g17 | 29.1% |

(1) only representative genes from *Oxytricha* are shown to minimize redundancy in this table. (2) Note that these sequences may not be a 1:1 orthologs, as phylogenies based on the nucleotide sequences of these proteins typically cluster the *Oxytricha* H3.1/3.2 together.

**Known protein domains are conserved between *Stylonychia* and *Oxytricha***

As we have previously mentioned, there is significant sequence similarity betwen *Stylonychia* and *Oxytricha*, and from our analyses of macronuclear genome properties of *Stylonychia* and *Oxytricha* it can also be seen that these two species are generally quite similar. We set out to determine if there were outstanding qualitative differences between the types of predicted proteins in *Stylonychia* and *Oxytricha*, since this might point to important functional differences between these species. Since the sensitivity of the method used to annotate proteins strongly influences which proteins differ between predicted proteomes, we decided to use sensitive HMMER3 ([Eddy 2013](#_ENREF_25)) searches of Pfam to investigate differences in the domain complements of *Stylonychia* and *Oxytricha*.

Selecting only matches with e-values < 1e-6, we found 2495 domains in common between *Stylonychia* and *Oxytricha*. *Stylonychia* had 155 domain matches missing in *Oxytricha*, and *Oxytricha* had 157 domain matches missing in *Stylonychia*. Some of the species-specific matches appear to be due to erroneous or missing gene predictions, or the arbitrary e-value cut-off chosen. For instance, as judged by TBLASTN searches, of the three ribosomal domains detected in *Stylonychia* but not *Oxytricha*, Ribosomal_L33 and Ribosomal_L44 are missing in *Oxytricha* due to gene prediction failures, and Ribosomal_L37 is present in *Oxytricha*, but falls just below our e-value cut-off. We therefore conclude that *Stylonychia* and *Oxytricha* proteins have similar known protein domain complements.
